# Disruption of *Chtf18* Causes Defective Meiotic Recombination in Male Mice

**DOI:** 10.1371/journal.pgen.1002996

**Published:** 2012-11-01

**Authors:** Karen M. Berkowitz, Aislinn R. Sowash, Lydia R. Koenig, Dawnette Urcuyo, Fahmida Khan, Fang Yang, P. Jeremy Wang, Thomas A. Jongens, Klaus H. Kaestner

**Affiliations:** 1 Department of Obstetrics and Gynecology, Drexel University College of Medicine, Philadelphia, Pennsylvania, United States of America; 2 Department of Biochemistry and Molecular Biology, Drexel University College of Medicine, Philadelphia, Pennsylvania, United States of America; 3 Department of Animal Biology, School of Veterinary Medicine, University of Pennsylvania, Philadelphia, Pennsylvania, United States of America; 4 Department of Genetics, School of Medicine, University of Pennsylvania, Philadelphia, Pennsylvania, United States of America; Cornell University, United States of America

## Abstract

CHTF18 (chromosome transmission fidelity factor 18) is an evolutionarily conserved subunit of the Replication Factor C-like complex, CTF18-RLC. CHTF18 is necessary for the faithful passage of chromosomes from one daughter cell to the next during mitosis in yeast, and it is crucial for germline development in the fruitfly. Previously, we showed that mouse *Chtf18* is expressed throughout the germline, suggesting a role for CHTF18 in mammalian gametogenesis. To determine the role of CHTF18 in mammalian germ cell development, we derived mice carrying null and conditional mutations in the *Chtf18* gene. *Chtf18*-null males exhibit 5-fold decreased sperm concentrations compared to wild-type controls, resulting in subfertility. Loss of *Chtf18* results in impaired spermatogenesis; spermatogenic cells display abnormal morphology, and the stereotypical arrangement of cells within seminiferous tubules is perturbed. Meiotic recombination is defective and homologous chromosomes separate prematurely during prophase I. Repair of DNA double-strand breaks is delayed and incomplete; both RAD51 and γH2AX persist in prophase I. In addition, MLH1 foci are decreased in pachynema. These findings demonstrate essential roles for CHTF18 in mammalian spermatogenesis and meiosis, and suggest that CHTF18 may function during the double-strand break repair pathway to promote the formation of crossovers.

## Author Summary

Meiosis is the specialized process of cell division during germ cell development that results in formation of eggs and sperm. Genetic exchange between maternal and paternal chromosomes occurs during meiosis in a process called homologous recombination, in which DNA double- strand breaks are made and then repaired to allow DNA crossovers to form. These are essential processes that keep homologous chromosomes joined until anaphase I and ensure proper chromosome segregation. Errors in meiotic recombination lead to chromosome mis-segregation and ultimately aneuploidy, an abnormal chromosome number. Although it is well known that defects in these processes contribute greatly to infertility, birth defects, and pregnancy loss in humans, their molecular basis is not well understood. We demonstrate here a *Chtf18* mutant mouse that exhibits subfertility and defects in meiotic recombination. Specifically, DNA double-strand breaks are incompletely repaired, DNA crossovers are significantly decreased, and homologous chromosomes separate during prophase I in *Chtf18*-null males. Our findings suggest roles for CHTF18 in DNA double-strand break repair and crossover formation, functions in mammals not previously known.

## Introduction

Precise chromosome segregation is crucial to ensure that germ cell development proceeds normally during meiosis, and that genetic information is accurately transmitted to the gametes. For chromosome segregation to proceed flawlessly during meiosis, homologous chromosomes must undergo several processes that allow them to pair and remain physically joined until anaphase I. The physical connections between homologous chromosomes are established by at least three different mechanisms. Sister chromatids are connected between arms and at centromeres by cohesion, a process mediated by cohesins, i.e. multiprotein complexes that are established during S-phase [1]–[6]. Different cohesin complexes exist depending on the cell type and stage, and each consists of at least four subunits, some of which are specific to meiosis [7], [8]. The physical connection between homologues also occurs by synapsis during meiotic prophase I, when homologous chromosomes pair through formation of a tripartite protein structure: the synaptonemal complex (for a review see [9]). Meiosis-specific cohesin complexes are believed to form a scaffold to which components of the synaptonemal complex can attach [10]. During synapsis, additional physical contacts occur at points of DNA crossover (chiasmata) through reciprocal recombination between nonsister chromatids (reviewed in [11], [12]). At the end of prophase I, the synaptonemal complex disassembles, but homologous chromosomes remain joined across sister chromatid arms and at centromeres. At anaphase I, dissolution of cohesion between sister chromatid arms and resolution of chiasmata allow homologous chromosomes to migrate away from the metaphase plate [13]. Although cohesion between sister chromatid arms is dissolved, cohesion at centromeres is preserved to keep sister chromatids connected until they segregate in anaphase II following attachment to the spindle [14]. Thus, cohesion and chiasmata between sister chromatid arms prevent homologues from separating prematurely [10], [11].

Maintenance of genome integrity is mediated in part by Replication Factor C- like complexes (RLCs) which function in DNA replication, chromosome cohesion, and the DNA damage checkpoint [15]. CTF18, a component of RLC-CTF18, was initially discovered in *Saccharomyces cerevisiae* [16]. In yeast, RLC-CTF18 is essential for establishment of sister chromatid cohesion and genome stability [15], [17], [18]. CTF18 is also crucial for germline development in the fruitfly. In *CTF18* mutant flies, termed *Cutlet*, a loss-of-function mutation causes failure of germline stem cells to proliferate normally, resulting in sterility [19]. Recently, studies of the human RLC-CTF18 (termed RLC-CHTF18) complex *in vitro* and in immortalized cell lines have demonstrated a role for CHTF18 in mammalian DNA replication [20]–[22]. Previously, we cloned and characterized *Chtf18*, the murine orthologue of *CTF18*. We showed that CHTF18 is expressed throughout the male and female germline of the mouse, suggesting a role for it in gametogenesis [23]. However, the role of CHTF18 in mammals has not been fully elucidated.

Here we report the crucial roles CHTF18 plays during male meiosis *in vivo*. Our data demonstrate that CHTF18 functions in mammalian spermatogenesis to ensure fertility in males. Our results also suggest a role for CHTF18 in male meiotic recombination, and that it may function in maintaining the linkage of homologous chromosomes during meiotic prophase I.

## Results

### Derivation of *Chtf18* ^−/−^ and *Chtf18* ^flox/flox^ mice

**Figure 1:**
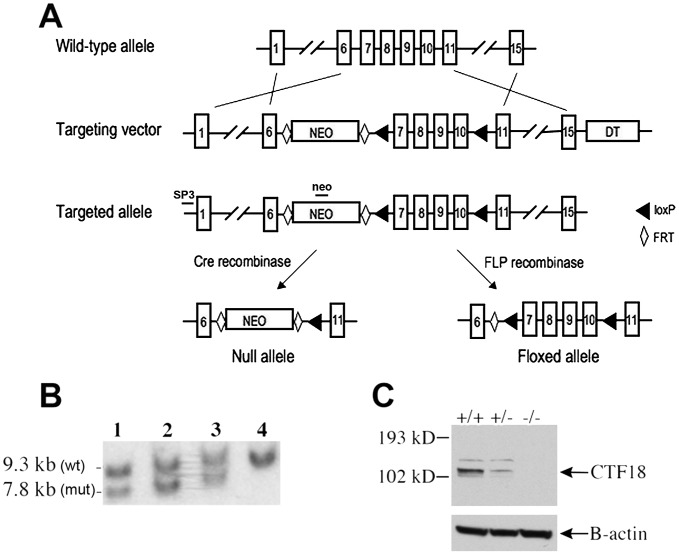
Derivation of *Chtf18* ^−/−^ and *Chtf18^flox/flox^* mice. (A) Gene targeting strategy (exons are not drawn to scale). LoxP sites are represented by triangles. Diamonds represent FRT sites. (B) Southern blot analysis of targeted ES cells (lanes 1, 2, and 3) and wild-type ES cells (lane 4). The probes used for Southern blot analysis are depicted as SP3 and neo. (C) Western blot analysis of testis protein extracts from *Chtf18*-null mice with CHTF18 antibody. A ß-actin specific antibody was used as a control.

CHTF18 is expressed throughout the male germline of the mouse, suggesting a role for it in spermatogenesis [23]. In order to study CHTF18 function *in vivo*, we employed gene targeting to derive *Chtf18* mutant mice. We constructed *Chtf18*-null and conditional alleles by homologous recombination in embryonic stem (ES) cells (Figure 1A). The mouse *Chtf18* gene consists of 22 exons spanning 8 kb of genomic DNA [23]. We chose to target exons 7–10, because these exons encode sequence motifs with high sequence similarity to Replication Factor C (RFC) (Figure 1A). These sequence motifs are called RFC boxes and are required for the function of RFC in yeast and in human cells [24]–[26]. Following electroporation and screening of 300 ES cell clones, five correctly targeted clones containing the *Chtf18^loxP^*-flanked (*Chtf18^flox^*) allele were identified (three are shown in Figure 1B). Cells from each of three clones were injected into mouse blastocysts and yielded 19 highly chimeric (>90%) male mice, which resulted in germline transmission of the targeted allele. Mice carrying this allele were then mated with transgenic Cre mice under the control of the E2A promoter [27]. The resulting heterozygotes were bred to homozygosity to generate *Chtf18* ^−/−^ mice. Absence of CHTF18 protein in *Chtf18* ^−/−^ testes was confirmed by Western blot analysis, indicating that this is a null allele (Figure 1C). To derive *Chtf18^flox/−^* TNAP-Cre mice, mice homozygous for the *Chtf18^flox^* allele were bred with transgenic Cre mice under the control of the germ cell-specific promoter of the tissue non-specific alkaline phosphatase (TNAP) gene [28], following FLP-mediated excision of the neomycin resistance cassette *in vivo* (Figure 1 and Figure S1).

### *Chtf18*-null mice are viable but smaller in body weight

**Figure 2:**
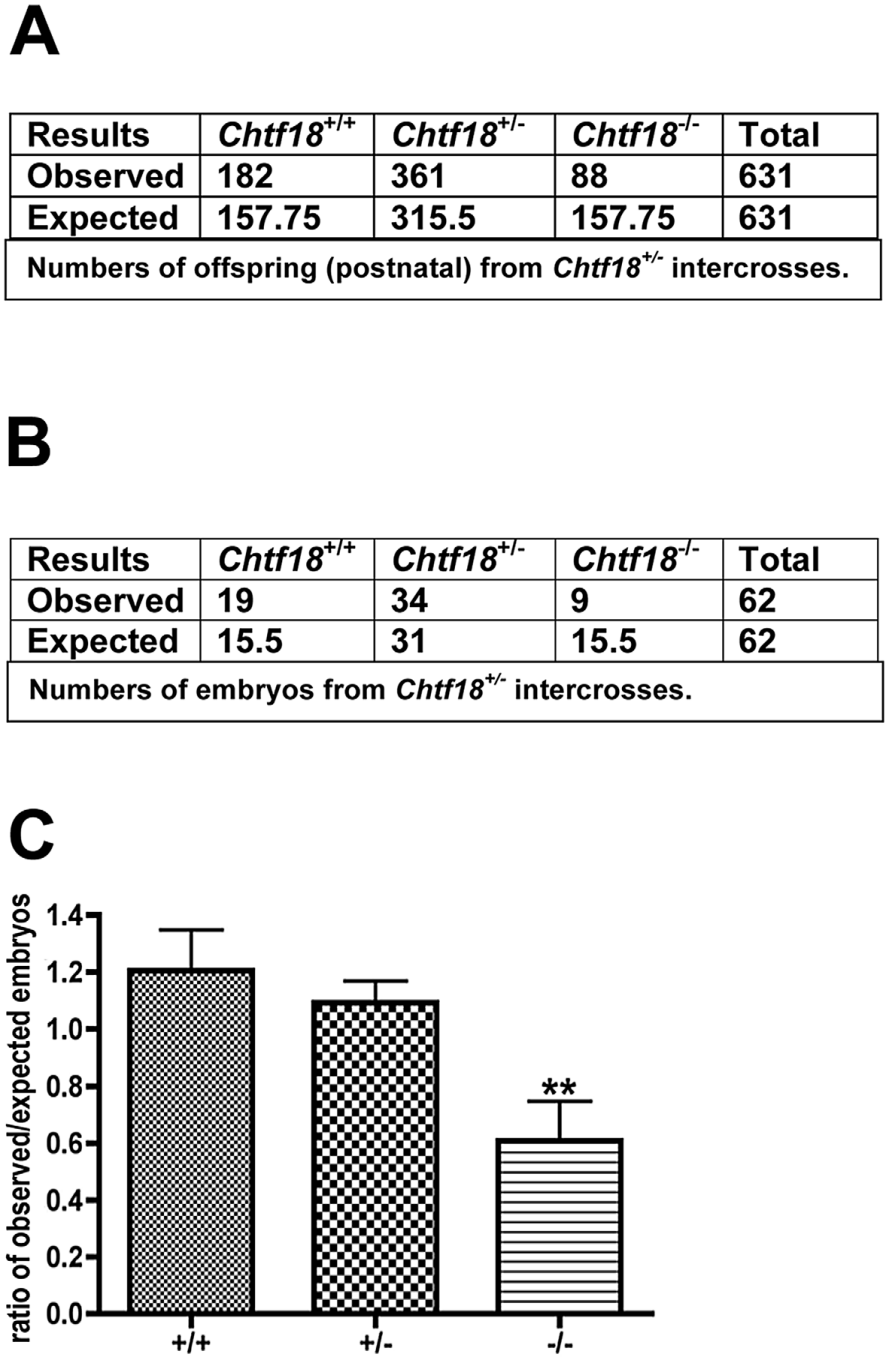
*Chtf18* ^−/−^ mice are born at submendelian ratios. Data collected from *Chtf18^+/−^* intercross matings. (A and B) Numbers of expected and observed *Chtf18* ^+/+^, *Chtf18* ^+/−^, and *Chtf18* ^−/−^ offspring and embryos (14.5–18.5 dpc), respectively. (C) Histograms showing observed/expected ratios of *Chtf18* ^+/+^, *Chtf18* ^+/−^, and *Chtf18* ^−/−^ embryos at 14.5–18.5 dpc. Values are the mean ratio ± SEM; p≤0.007 using ANOVA.

Although *Chtf18* ^−/−^ mice were viable with no overt defects, they were smaller in body weight (*Chtf18* ^−/−^ adult mean weight about 15% less than adult mean weight of wild-type), and were born at submendelian ratios (mean ratio of expected/observed embryos, *Chtf18* ^−/−^ 0.6, *Chtf18* ^+/+^ 1.2, *Chtf18^+/−^* 1.1, p<0.007 using ANOVA, Figure 2C). Data collected from *Chtf18^+/−^* intercross matings revealed approximately 50% of the expected number of *Chtf18* ^−/−^ offspring, compared to *Chtf18* ^+/+^ and *Chtf18^+/−^* offspring (Figure 2A). Data collected postnatally and from embryos at 14.5–18.5 dpc (Figure 2B and 2C) revealed virtually the same ratios of observed/expected for each genotype, and confirmed that these numbers were due to higher rates of death among *Chtf18* ^−/−^ mice during embryonic development.

### Impaired spermatogenesis, oligospermia, and decreased fertility in *Chtf18*-null males

**Figure 3:**
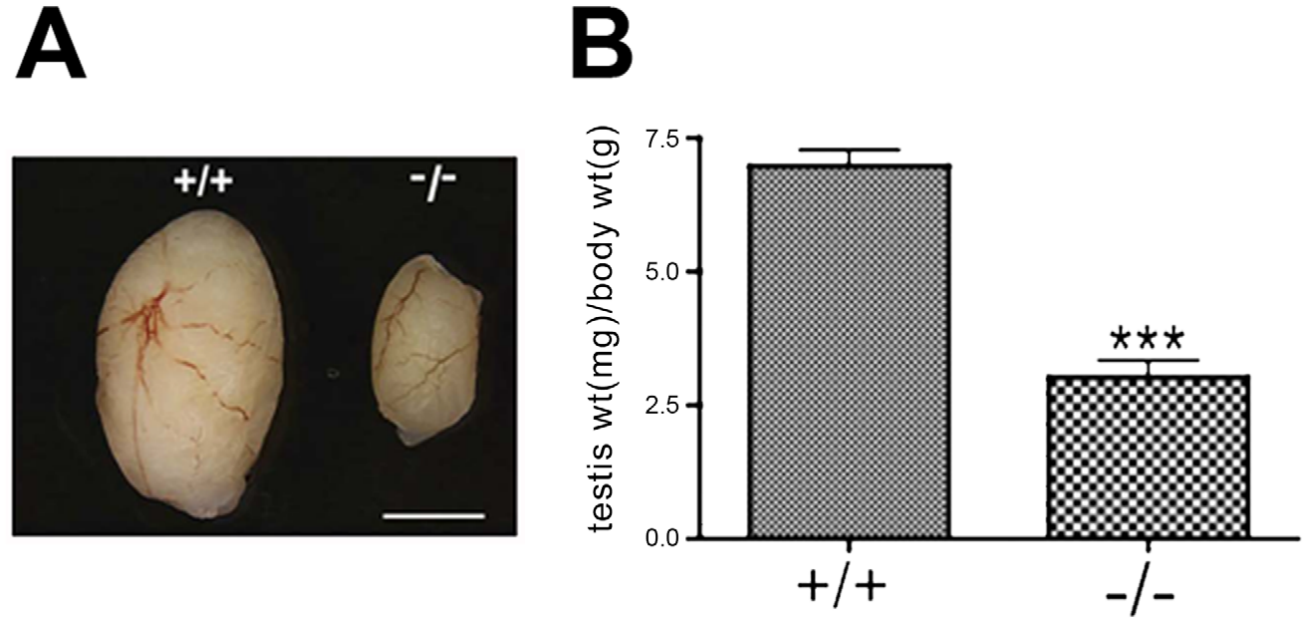
*Chtf18* ^−/−^ testes are significantly smaller than those of wild-type mice. (A) Dramatic size reduction in *Chtf18* ^+/+^ and *Chtf18* ^−/−^ adult testes. Scale bar represents 2 mm. (B) Histograms showing testis weight per body weight for *Chtf18* ^+/+^ and *Chtf18* ^−/−^ mice (mg/g, means ± SEM) *Chtf18* ^+/+^ 6.98±0.30, n = 9 mice; *Chtf18* ^−/−^ testes 3.03±0.32, n = 8 mice, P<0.0001 using the Student's t-test.

**Figure 4:**
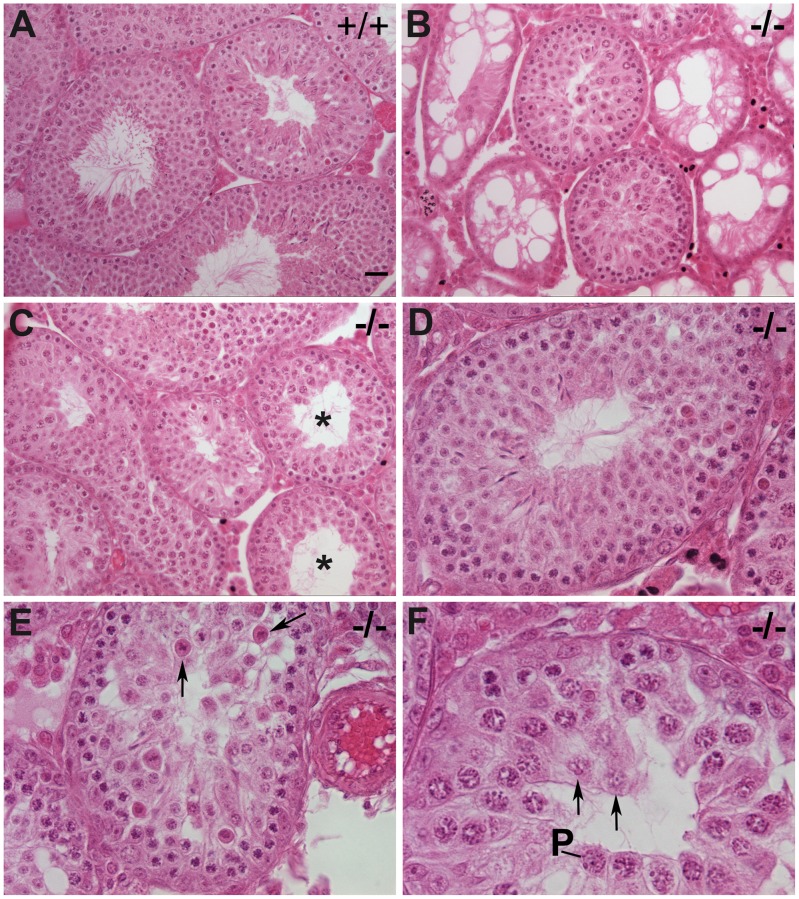
Impaired spermatogenesis in *Chtf18* ^−/−^ mice. Seminiferous tubules stained with hematoxylin and eosin from 6–12 week old wild-type (A) and *Chtf18* ^−/−^ testes (B–F) are shown. *Chtf18* ^−/−^ tubules show a range of abnormalities. A paucity of spermatids and spermatozoa (asterisks in C) or almost complete absence of spermatogenic cells and large vacuoles (B) are seen, although a few *Chtf18* ^−/−^ tubules demonstrate almost normal morphology (D). Disorganized testes morphology and aberrant spermatogenic cells (E and F) are also seen. Spermatogenic cells appear to be in pachynema, P, are located at the lumen, and arrows in E and F indicate aberrant cells in *Chtf18* ^−/−^ seminiferous tubules. Scale bar is 25 µm.

Although *Chtf18* ^−/−^ mice appeared grossly normal but smaller in body weight, testes of *Chtf18* ^−/−^ males were significantly smaller (testis weight per body weight for *Chtf18* ^+/+^ and *Chtf18* ^−/−^ mice mg/g, means ± SEM, *Chtf18* ^+/+^ 6.98±0.30, n = 9 males; *Chtf18* ^−/−^ 3.03±0.32, n = 8 males, P<0.0001 using the Student's *t*-test, Figure 3A and 3B) and morphologically abnormal compared to those of control littermates (Figure 4). Although seminiferous tubules of *Chtf18* ^−/−^ and wild-type testes contained the complete spectrum of spermatogenic cells, including spermatogonia, spermatocytes, spermatids, and spermatozoa, indicating that there is not a block in spermatogenesis at any specific stage, the cells demonstrated a range of abnormalities. *Chtf18* ^−/−^ seminiferous tubules contained large multinucleated and aberrant-appearing spermatogenic cells, while others were almost devoid of spermatogenic cells (Figure 4B), although a few tubules showed almost normal morphology (Figure 4D). In addition, the stereotypical arrangement of spermatogenic cells demonstrating the orderly progression of spermatogenesis within seminiferous tubules appeared absent in most *Chtf18* ^−/−^ tubules, suggesting that spermatogenesis is impeded or disrupted (Figure 4E and 4F).

**Figure 5:**
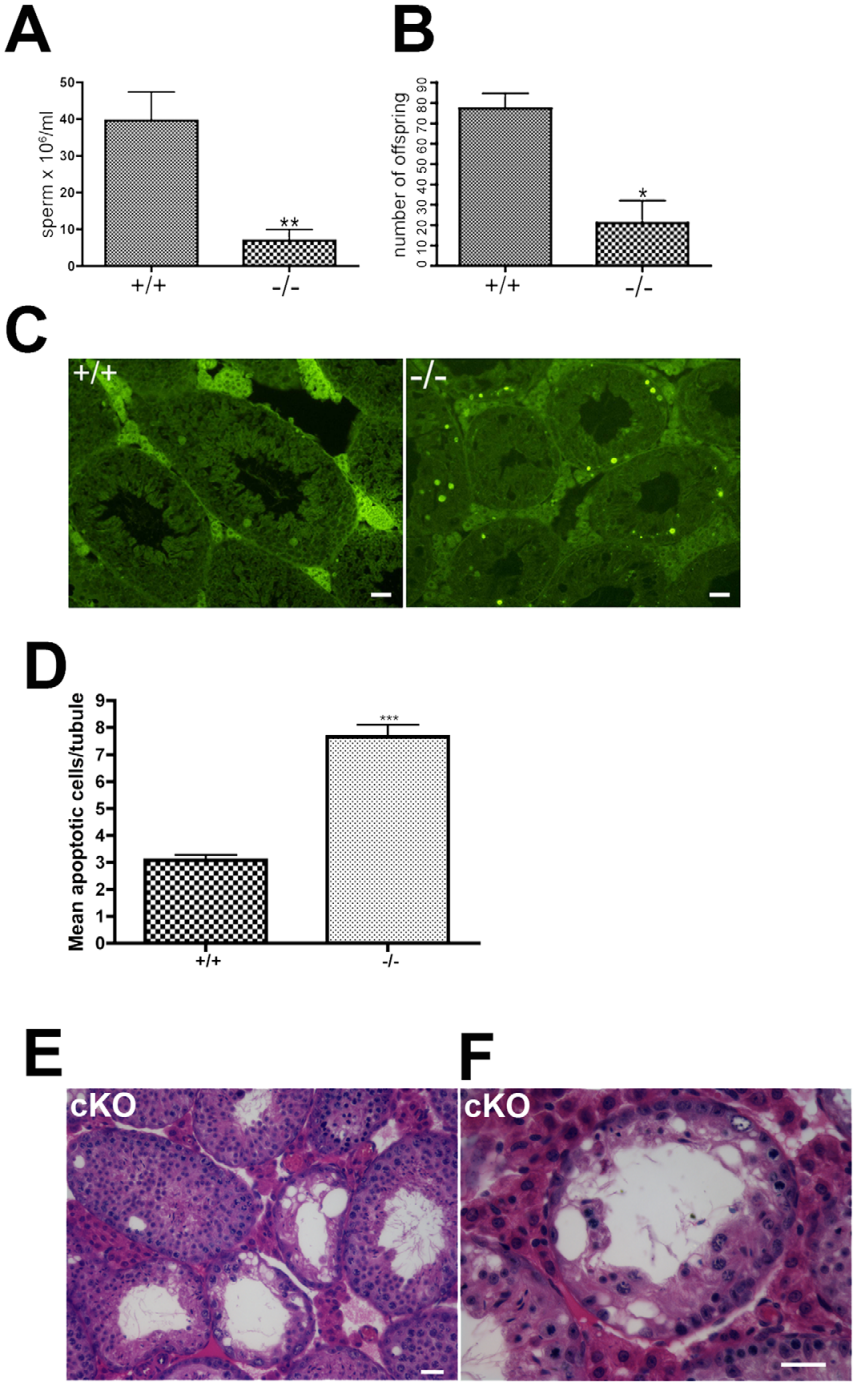
Oligospermia and decreased fertility in *Chtf18*-null mice. (A) Histograms showing caudal epididymal sperm concentrations from 10–12 week old *Chtf18* ^−/−^ mice and *Chtf18* ^+/+^ controls. Values are the number of sperm ×10^6^ per ml, means ± SEM, *Chtf18* ^+/+^ 39.60±7.71, n = 9 mice; *Chtf18* ^−/−^ 6.90±3.04, n = 8 mice, P≤0.002 using Student's t-test (B) Histograms showing the number of offspring from wild-type females bred with *Chtf18* ^−/−^ and *Chtf18* ^+/+^ male mice over a 5 month period. Each male was paired with two wild-type females (number of offspring from 12 pairs of wild-type females mated with *Chtf18* ^+/+^ and *Chtf18* ^−/−^ males, means ± SEM, *Chtf18* ^+/+^ 77.33±7.36, n = 3 males; *Chtf18* ^−/−^ 21.00±10.97, n = 3 males, P≤0.0130 using Student's t-test) (C) TUNEL staining of seminiferous tubule sections from three *Chtf18* ^−/−^ and three wild-type 7 week old mice demonstrates that apoptotic cells are increased in *Chtf18* ^−/−^ tubules. Scale bar is 25 µm. (D) Histograms showing mean apoptotic cells per tubule ± SEM; wild-type (+/+) 3.083±0.197; N = 145 vs. *Chtf18* ^−/−^ (−/−) 7.658±0.477; N = 155; p<0.0001 (E and F) Tubules from 13–14 week old *Chtf18^flox/−^*; TNAP Cre (cKO) mice stained with hematoxylin and eosin demonstrate a morphological phenotype that is indistinguishable from *Chtf18* ^−/−^ tubules (Figure 4). Scale bars are 25 µm and 50 µm for E and F, respectively.

To determine the physiologic consequences of the disruption seen in seminiferous tubules of *Chtf18* ^−/−^ mice, we quantified the number of sperm recovered from the caudal region of the epididymis. We found that the sperm counts of *Chtf18* ^−/−^ mice were reduced more than 5-fold compared to those of wild-type males (Figure 5A). To evaluate the impact of this severe oligospermia on fertility of *Chtf18* ^−/−^ males, we mated *Chtf18* ^−/−^ or wild-type males with pairs of wild-type females over a period of five months. As expected from the low sperm counts, we found that *Chtf18* ^−/−^ males are subfertile compared to wild-type controls (Figure 5B). Loss of *Chtf18* leads to oligospermia and not meiotic arrest, since some mature spermatids are indeed produced. Thus, the phenotype results in subfertility and not sterility in males. To assess whether apoptosis was an underlying cause of the paucity of spermatogenic cells in *Chtf18* ^−/−^ seminiferous tubules, we performed TUNEL assays. We found an increased number of apoptotic cells in *Chtf18* ^−/−^ tubules (mean number of apoptotic cells per seminiferous tubule, 7.66 and 3.08 for three *Chtf18* ^−/−^ and three wild-type adult males, respectively, p<0.0001, Figure 5C and 5D).

Because CHTF18 is expressed in both the somatic and germ cell lineages in the testes, we wanted to determine whether *Chtf18* is required specifically in germ cells. To this end, we derived *Chtf18* ^flox/−^ TNAP Cre mice (cKO), in which *Chtf18* is deleted only in germ cells (Figure S1). Several studies have demonstrated the use of the TNAP Cre transgenic mouse to effect highly specific and efficient germ cell-specific deletion [28]–[32]. As shown in Figure 5E and 5F, the morphological phenotype of affected cKO spermatogenic cells is indistinguishable from those seen in *Chtf18* ^−/−^ testes, suggesting that *Chtf18* is required cell-autonomously in germ cells. While pre-meiotic effects cannot be ruled out, somatic effects of the testes can be excluded since TNAP is not expressed in these cells.

**Figure 6:**
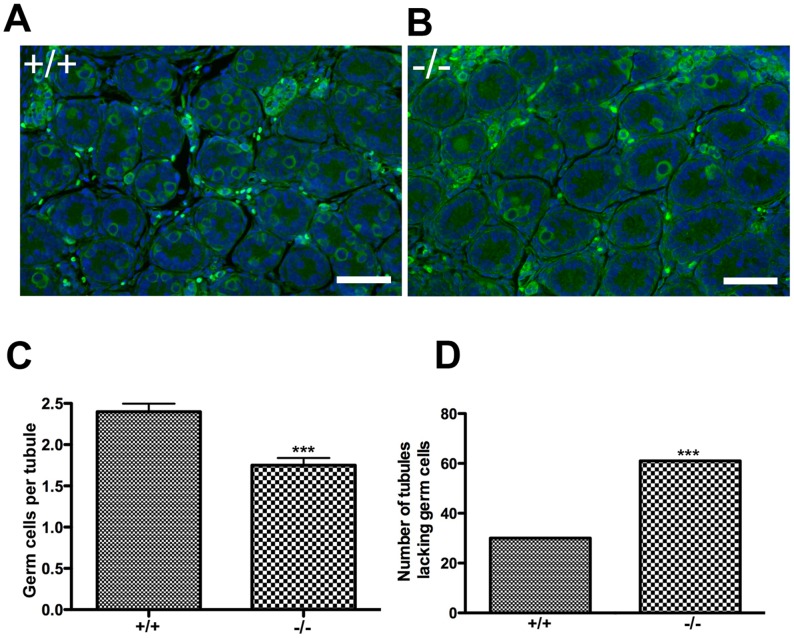
The number of prospermatogonia is decreased in *Chtf18* ^−/−^ neonatal testes. (A and B) Testis sections from postnatal day 3 *Chtf18*-null and wild-type males were stained with anti-MVH (green) and DAPI (blue). Scale bars are 50 µm. (C) Histogram showing the mean number of MVH-positive cells per seminiferous tubule ± SEM; wildtype (+/+) 2.399±0.097; N = 278 tubules and *Chtf18* ^−/−^ (−/−) 1.750±0.090; N = 280 tubules; p<0.0001 using Student's t-test (D) Histogram showing the number of seminiferous tubules completely lacking prospermatgonia in *Chtf18* ^−/−^ and wild-type testes; wild-type (+/+) 30, N = 278 and *Chtf18* ^−/−^ 61, N = 280, p = 0.0005 using Fisher's exact test.

*CTF18* mutant flies (called *Cutlet*) exhibit cessation of germline stem cell proliferation in mitotic stages of amplification [19]. Therefore, we speculated that a defect in establishment or maintenance of the early spermatogonial pool (prospermatogonia) might contribute to the paucity of germ cells seen in *Chtf18* ^−/−^ tubules. To evaluate this population of cells we stained postnatal day 3 (P3) testis sections from *Chtf18* ^−/−^ compared to wild-type tubules with an antibody to mouse vasa homolog (MVH), a germ cell-specific marker. Although we did not see a progressive loss of spermatogonial cells in *Chtf18* ^−/−^ tubules, we found that the number of prospermatogonia was significantly decreased in seminiferous tubules of *Chtf18* ^−/−^ compared to wild-type mice (mean number of germ cells per seminiferous tubule, 2.399 and 1.750 for four males each, respectively, p<0.0001, Student's t-test, Figure 6A–6C). In addition, the number of tubules completely lacking prospermatgonia was significantly greater in *Chtf18* ^−/−^ than wild-type testes (61, N = 280 tubules and 30, N = 278 tubules for four males each, respectively, p = 0.0005, Fisher's exact test, Figure 6D). These data suggest that that there is a defect in the early germ cell lineage of *Chtf18* ^−/−^ males.

### Homologous chromosomes separate prematurely in *Chtf18*-null mice

**Figure 7:**
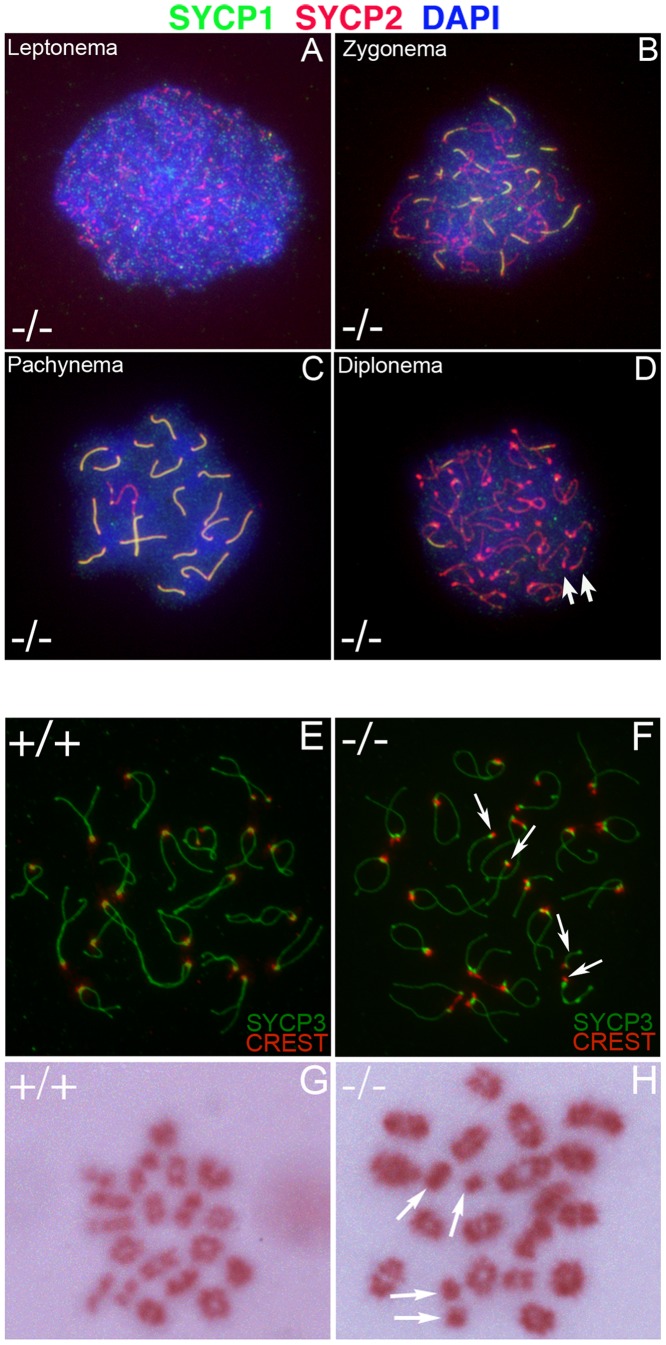
Formation of univalent chromosomes in *Chtf18* ^−/−^ spermatocytes. Meiotic chromosome spreads from *Chtf18* ^−/−^ juvenile males at postnatal day 21 were stained by immunofluorescence with anti-SYCP1 serum (green), anti-SYCP2 serum (red), and DAPI (blue) (A–D). SYCP1 localizes to central elements of the synaptonemal complex and SYCP2 localizes to the axial/lateral elements of the synaptonemal complex. The leptone, zygotene, pachytene, and diplotene stages of prophase I *Chtf18* ^−/−^ spermatocyte (−/−) are shown (A, B, C, and D respectively). Arrows in D reveal the presence of univalent chromosomes in the diplotene stage of prophase I. Wild-type and *Chtf18* ^−/−^ spermatocytes from juvenile males at postnatal day 21 (E and F, respectively) were stained with CREST autoimmune serum, which stains centromeres, and anti-SYCP3, which stains the axial/lateral elements of the synaptonemal complex. Arrows in F indicate univalent chromosomes in a *Chtf18* ^−/−^ diplotene spermatocyte. Metaphase I spread analysis of spermatocyte nuclei from juvenile males at postnatal day 21 stained with Giemsa (G and H). Twenty bivalent chromosomes are present in the wild-type metaphase I spermatocyte (G). A *Chtf18* ^−/−^ metaphase I spermatocyte (H) demonstrates the presence of four univalent chromosomes (arrows in H).

To begin to identify the molecular basis underlying the observed defects in *Chtf18* ^−/−^ mice, we examined meiosis. Surface spread analysis of *Chtf18* ^−/−^ spermatocytes, immunostained with anti-SYCP1 and anti-SYCP2 antibodies which label central and axial/lateral elements of the synaptonemal complex, were used to evaluate the progression of meiotic prophase I. We found that the leptotene and zygotene stages of prophase I progressed normally in *Chtf18* ^−/−^ spermatocytes as demonstrated by accumulation of SYCP1 and SYCP2 on homologues (Figure 7A and 7B). Synapsis of homologous chromosomes during the pachytene stage was also normal as demonstrated by the complete co-localization of SYCP1 and SYCP2 on autosomes of 105 *Chtf18* ^−/−^ pachytene spermatocytes compared to 100 pachytene wild-type cells (Figure 7C). However, examination of the diplotene stage of prophase I revealed the presence of separated homologues, consistent with univalent chromosomes (Figure 7D). To confirm the presence of univalent chromosomes we used CREST autoimmune serum, which stains centromeres, and anti-SYCP3, which stains the axial/lateral elements of the synaptonemal complex (Figure 7E and 7F). We quantified the number of CREST foci on homologues in *Chtf18*-null compared to wild-type spermatocytes. Univalent chromosomes were counted in diplotene spermatocytes containing greater than 21 CREST foci on separated homologues. We found that 42% of *Chtf18* ^−/−^ diplotene spermatocytes (66 cells counted in four males) contained univalent chromosomes (Figure 7F), while no univalent chromosomes were seen in wild-type diplotene spermatocytes (70 cells counted in three males, Figure 7E). In affected *Chtf18* ^−/−^ diplotene spermatocytes, we found two or more univalent chromosomes. These data are consistent with premature separation of homologous chromosomes during prophase I and not asynapsis because pairing of homologues and chromosomal synapsis through the pachytene stage in *Chtf18* ^−/−^ mice were normal.

### Early disjunction of *Chtf18*-null homologues persists into metaphase I

Next we performed metaphase I spread analysis of spermatocytes to determine whether univalent chromosomes persist after synaptonemal complex dissolution at the end of prophase I (Figure 7G and 7H). Univalent chromosomes (as many as six) were present in 20% (35 cells counted) of *Chtf18* ^−/−^ metaphase I spermatocytes (Figure 7H), a number that is highly significant in light of the fact that such chromosomes were not observed in spermatocytes of wild-type males (50 cells counted). These findings reveal that homologues in *Chtf18* ^−/−^ spermatocytes separate prematurely during meiotic prophase I and that the defect persists through metaphase I, resulting in formation of univalent chromosomes.

### Meiotic recombination is defective in *Chtf18*-null spermatocytes

In order to evaluate progression of meiotic recombination and DNA double-strand break (DSB) repair, we performed immunostaining with antibodies to γH2AX and RAD51, markers of DSB repair. Following formation of DSBs during leptonema, γH2AX is found along chromatin of both autosomes and sex chromosomes during normal meiotic progression [33]. γH2AX staining decreases during prophase I as DSBs are repaired until it is confined to the sex body, a region containing both the X and Y chromosomes, during pachynema. RAD51 foci appear as early meiotic recombination nodules, and they are abundant throughout prophase I. The number of RAD51 foci peaks in leptotema and early zygonema, and decreases in late pachynema as DSBs are repaired [34]–[36].

**Figure 8:**
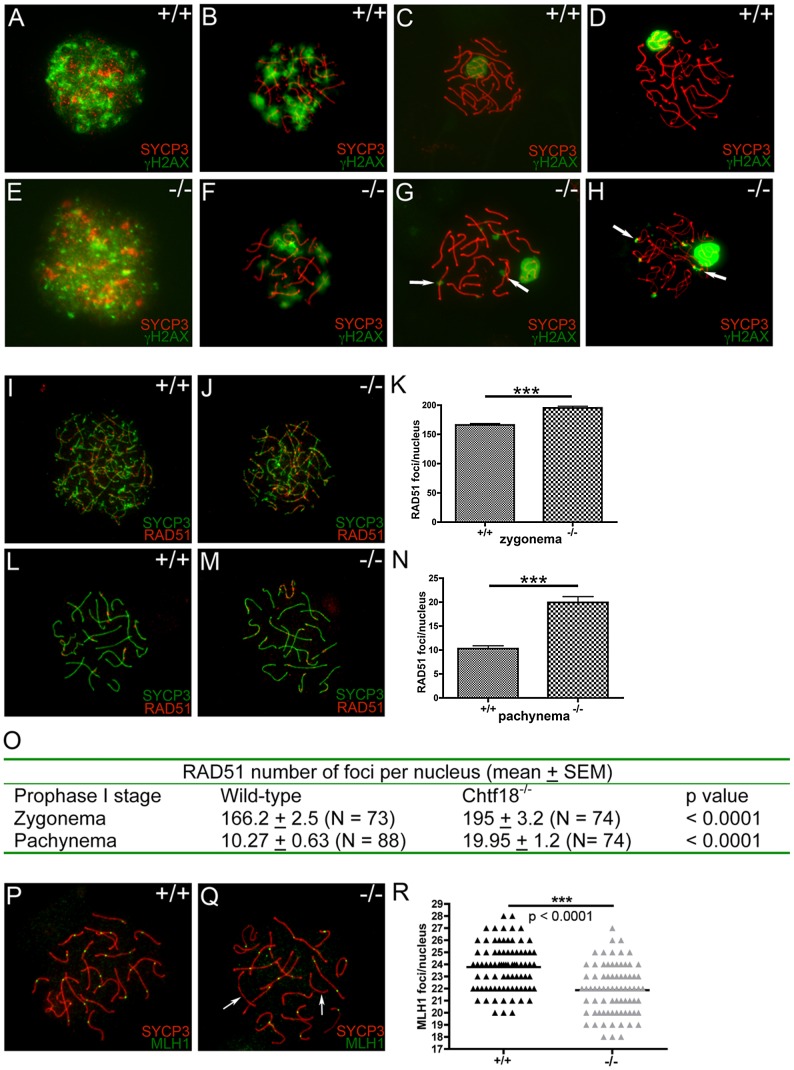
Meiotic recombination is defective in *Chtf18* ^−/−^ spermatocytes. Meiotic chromosome spreads from wild-type (A–D) and *Chtf18* ^−/−^ (E–H) juvenile males at postnatal day 14 or 21 were stained with anti-SYCP3 (red) and γH2AX (green). A & E represent leptonema; B & F represent zygonema; C & G represent pachynema; D & H represent diplonema. Arrows in G and H show persistence of γH2AX in *Chtf18* ^−/−^ spermatocytes. Meiotic chromosome spreads from wild-type (I and L) and *Chtf18* ^−/−^ (J and M) males were stained with anti-SYCP3 (green) and anti-RAD51 (red). I and J represent zygonema; L and M represent pachynema. RAD51 focus counts per nucleus in zygonema (K and O) and pachynema (N and O) are shown. Meiotic chromosome spreads from wild-type and *Chtf18* ^−/−^ spermatocytes were stained with anti-SYCP3 (red) and anti-MLH1 (green) (P and Q). Arrows indicate homologues without MLH1 foci. MLH1 focus counts per nucleus are shown in R.

In both wild-type and *Chtf18*-null spermatocytes meiotic recombination initiated normally as demonstrated by the appearance of γH2AX during leptonema (Figure 8A and 8E). Although γH2AX staining decreased similarly in both wild-type and *Chtf18*-null spermatocytes during zygonema (Figure 8B and 8F) and became restricted to the sex body in wild type spermatocytes in pachynema and diplonema (Figure 8C and 8D), it persisted on the autosomes of *Chtf18* ^−/−^ spermatocytes into pachynema and diplonema (Figure 8G and 8H). This suggests that DSBs are formed but not repaired efficiently in the absence of CHTF18. Immunostaining with anti-RAD51 showed normal deposition of RAD51 on wild-type homologues in zygonema and pachynema (Figure 8I and 8L), but revealed the persistence of meiotic recombination nodules in *Chtf18*-null spermatocytes into zygonema and pachynema (Figure 8J and 8M). While the number of RAD51 foci decreased in wild-type spermatocytes by pachynema, *Chtf18*-null spermatocytes maintained a significantly greater number (mean number of RAD51 foci per nucleus, 10.27 and 19.95, in four control and four mutant males, respectively, p<0.0001, Figure 8N and 8O). DSB repair also appeared to be delayed as suggested by a significantly greater number of RAD51 foci detected on *Chtf18*-null compared to wild-type homologues during zygonema (mean number of RAD51 foci per nucleus, 195.2 and 166.2 for four *Chtf18* ^−/−^ and four wild-type males, respectively, p<0.0001, Figure 8K and 8O), indicating that the early stages of meiotic recombination are affected by loss of *Chtf18*.

Next we used an antibody to the mismatch repair protein, MLH1, to evaluate DSB repair and formation of meiotic crossovers in *Chtf18* ^−/−^ spermatocytes. MLH1 localizes to meiotic nodules that are believed to be the sites where chiasmata form [37], [38], and resolution of chiasmata is necessary for homologue disjunction. While each homologue should have at least one crossover (MLH focus), we found a slight but statistically significant decrease in the number of MLH1 foci in *Chtf18* ^−/−^ compared to wild-type spermatocytes (21.87 and 23.77 for four *Chtf18* ^−/−^ and four wild-type males, respectively, p<0.0001, Figure 8Q and 8R). In addition, 16.9% of *Chtf18* ^−/−^ spermatocytes contained at least one autosome that completely lacked a MLH1 focus (N = 83 late pachytene cells) compared to 3.4% of wild-type spermatocytes (N = 87 late pachytene cells). Analysis excluding these cells revealed that the average number of MLH1 foci was still significantly decreased in *Chtf18* ^−/−^ spermatocytes (that did not lack foci) compared to wild-type spermatocytes (22.17 and 23.82 for four *Chtf18* ^−/−^ and four wild-type males respectively, p<0.0001, Figure S2). Since each homologue normally forms at least one crossover (the obligate CO), these data are consistent with fewer *Chtf18* ^−/−^ spermatocytes (i.e. those not lacking MLH1 foci) containing autosomes with two or more MLH1 foci (instead of one) compared to wild-type spermatocytes. These findings indicate that the meiotic recombination defects seen in *Chtf18* ^−/−^ spermatocytes persist well into the late stages of meiotic recombination, and suggest a role for *Chtf18* in facilitating normal rates of crossover during prophase I. In addition, the presence of univalent chromosomes in *Chtf18*-null diplotene and metaphase I spermatocytes (Figure 7) is due, at least in part to defective crossover formation at the pachytene stage.

## Discussion

We derived mice lacking CHTF18, an evolutionarily conserved protein that is crucial for fertility in the fruitfly, and essential for accurate chromosome segregation in yeast. We demonstrated that spermatogenesis is severely disrupted and fertility is significantly impaired in *Chtf18*-null males.

*Chtf18* is the murine orthologue of CTF18, a subunit of the replication factor C-like complex (RLC), RLC-CTF18, which consists of seven subunits (CTF18-CTF8-DCC1-RFC2-RFC3-RFC4-RFC5), and was initially discovered in *Saccharomyces cerevisiae* [16]. During DNA replication CHTF18 protein forms a complex with other RFC components to load PCNA (proliferating cell nuclear antigen; a replication fork protein essential for DNA replication) onto DNA. Studies in budding yeast have shown that CTF18 also functions in homologous recombination and DSB repair [39]. Loss of *CTF18* in yeast results in improper establishment of sister chromatid cohesion, genetic instability, and aneuploidy [15], [17], [18]. RLC-CTF18 seems to couple DNA replication with sister chromatid cohesion because it is recruited to the replication fork in response to replication arrest [40], but the way in which this occurs is unknown. Consistently, RLC-CTF18 is implicated in the replication checkpoint and functions as an efficient unloader of PCNA in *S. cerevisiae* [41], [42]. Moreover, CTF18 stabilizes replication forks to facilitate sister chromatid cohesion in *Schizosaccharomyces pombe* [43]. As mentioned above in the fruit fly, *CTF18* (called *Cutlet*) is necessary for fertility [19]. In humans, formation of the RLC-CHTF18 complex *in vitro* and in cell lines suggests a role for CHTF18 in mammalian DNA replication; human RLC-CHTF18 interacts with proliferating cell nuclear antigen and bound chromatin preferentially during S phase [20]–[22]. Recently, RLC-CHTF18 was shown to be necessary for the speed of DNA replication fork progression and efficient acetylation of cohesin in human epithelial cells, important processes that are necessary for continued advancement of DNA synthesis [44]. Thus, the functions of RLC-CTF18 appear to be conserved among eukaryotes.

Our data reveal that homologous chromosomes separate prematurely during meiosis I in *Chtf18* ^−/−^ spermatocytes. While homologous chromosomal pairing and synapsis are complete during pachynema, as revealed by deposition and co-localization of SYCP1 and SYCP2 (Figure 7C), univalent chromosomes are detected in diplonema of prophase I and in metaphase I in mutant spermatocytes (Figure 7F and 7H). Our findings suggest that the pachytene checkpoint is not activated in *Chtf18* ^−/−^ spermatocytes, since spermatocytes progress beyond pachynema. Meiotic recombination is initiated and the early steps appear to proceed normally in *Chtf18*-null homologues, but DNA double-strand break repair (DSB) repair and crossover formation are defective. This is indicated by persistence of both γH2AX and RAD51, and decreased MLH1 foci number during prophase I. In addition, DSB repair appears to be delayed as suggested by a significantly greater number of RAD51 foci detected on *Chtf18*-null homologous chromosomes during zygonema (Figure 8J, 8K, and 8O). These data suggest that CHTF18 plays crucial roles in mammalian meiosis and that CHTF18 may function in preventing early homologue disjunction. These observations are consistent with a role for CHTF18 in maintaining homologue linkage and possibly in DSB repair. CHTF18 may prevent homologue disjunction through a mechanism that affects DSB repair and/or crossover formation. Homologue disjunction during anaphase I normally occurs with resolution of chiasmata, which necessitates removal of cohesin from chromosome arms distal to chiasmata but not at centromeres [45]–[47]. Persistence of DSBs seen in pachynema and diplonema may arise due to DSBs occurring before meiosis and not as a result of SPO-11 action during early prophase I. However, it is not likely a major contributing defect since synapsis of homologues occurs normally in *Chtf18*-null spermatocytes. The possibility of pre-meiotic defects during the spermatogonial stages of spermatogenesis cannot be excluded. CHTF18 protein is expressed in all stages of developing germ cells in adult males, and in fetal male germ cells from 13.5 through 15.5 dpc [23], and the number of prospermatogonia is significantly decreased in *Chtf18*-null compared to wild-type neonatal seminiferous tubules. Although the decreased number of prospermatogonia must originate during establishment or proliferation of the primordial germ cell population, there does not appear to be a defect in spermatogonial stem cell renewal in adult mutant mice. Adult *Chtf18*-null seminiferous tubules do not progressively lose spermatogonia as seen in the *Plzf* mutant mouse [48]. The defect in establishment or proliferation of prospermatgonia in *Chtf18*-null males is not as severe as that observed in the germline of *Drosophilia CTF18* mutants (termed *Cutlet*). In *Cutlet* mutant ovaries there is cessation of germline stem cell proliferation, and few if any egg chambers are formed, leading to sterility [20]. *Cutlet* mutant flies also exhibit eye and wing defects, but these defects are relatively mild and the adult organs appear to function normally. *Cutlet* mutations result in both decreased cellular proliferation and increased apoptosis in affected tissues, and studies implicate Cutlet as an accessory factor for DNA replication [19]. Similarly, *Chtf18* ^−/−^ mice are healthy but smaller in size than wild-type controls, suggesting a role for CHTF18 in cellular proliferation and DNA replication of somatic cells in mammals. The milder phenotype of early germ cell defects observed in *Chtf18* mutant mice compared to *Cutlet* mutant flies suggests a non-essential but more specialized role for CHTF18 in mammalian gametogenesis.

Premature homologue disjunction and a defect in DSB repair in *Chtf18* ^−/−^ spermatocytes are consistent with cohesion-dependent and cohesion-independent mechanisms. Cohesion is mediated by cohesin complexes, which form a ring-like structure and embrace chromatin fibers of sister chromatids from DNA replication until their separation during anaphase [5]. Segregation of homologues during meiosis I is elicited by loss of cohesin complexes along chromosome arms distal to chiasmata [49]. During meiosis, cohesin complexes are necessary for establishing and maintaining cohesion between sister chromatids, and for synapsis and recombination between homologous chromosomes [50]. DSB repair during meiosis also requires cohesion between sister chromatid arms to be maintained [45]. Although the exact role cohesion plays in homologous recombination and DSB repair during meiosis is not known, a recent study in *Caenorhabditis elegans* reveals that meiotic cohesin promotes DSB processing and recruitment of DNA damage checkpoint proteins early in the DNA damage response. Absence of cohesin from meiotic chromosomes causes loss of chiasmata and the persistence of DSBs with accumulation of recombination intermediates [51]. Therefore, it is possible that CHTF18 is involved in both cohesion action and its effects on DSB repair during meiosis. However, a role for CHTF18 in maintenance of homologous chromosome linkage in mammals has not been shown previously. In both budding yeast and vertebrates efficient repair of DSBs by homologous recombination relies on the ability of cohesin complexes to mediate sister chromatid cohesion, and cohesin complexes accumulate on chromatin at DSBs [52]–[56]. While it has been shown that cohesin accumulates at sites of DSBs in mitotically dividing mammalian cells [57], it is not known whether cohesin complexes can be loaded onto chromosomes of meiotic cells after S phase [8] in mammals. CHTF18 has been shown to interact with the cohesin complexes in human immortalized cells [58], [59]. Thus, CHTF18 may preserve homologue linkage by maintaining cohesion between sister chromatid arms that was established prior to S phase; this could occur by interaction of CHTF18 with cohesin complexes loaded at sites of DSBs during meiosis, by a crossover mechanism, or by a combination of both these mechanisms.

Studies have provided evidence that cohesion is directly coupled to DNA replication by physical interaction between cohesin proteins and proteins involved in DNA replication at the replication fork [60]. Possible mechanisms of replication-dependent sister chromatid cohesion have been provided by studies in yeast and human cell lines [20]–[22], [40]–[43]. Recent studies in yeast have led to a model in which RLC-CTF18 associates with chromosomes to regulate PCNA and establish sister chromatid cohesion at the replication forks [40]–[43]. Thus, CHTF18 may interact with cohesin complexes at the replication fork. While prior studies have demonstrated a clear role for CHTF18 in DNA replication and establishment of sister chromatid arm cohesion, our data suggest a role for CHTF18 in promoting crossover formation through a possible interaction with cohesin complexes. Our data also suggest a role for CHTF18 in DSB repair and ensuring a wild-type number of crossovers in spermatocytes. Interestingly, approximately 17% of *Chtf18*-null spermatocytes in late pachynema contain at least one autosome that completely lacks a MLH1 focus (i.e. the majority of *Chtf18*-null spermatocytes have autosomes that each contain at least one MLH1 focus), yet homologues separate prematurely in 42% of *Chtf18*-null spermatocytes during diplonema. A possible explanation is that one crossover per homologue in the presence of impaired sister chromatid cohesion does not provide enough stability to prevent premature separation of homologues in *Chtf18*-null spermatocytes. This would suggest that in addition to its canonical function during DNA replication, CHTF18 acts as a cohesion factor to facilitate crossover formation during meiotic recombination. It is unclear exactly how CHTF18 might interact with cohesion factors. It is possible that CHTF18 affects this process during pre-S phase and/or during the DSB repair pathway in meiosis. Both chiasmata and cohesion between sister chromatid arms distal to chiasmata prevent homologues from separating prematurely [10], [11] and *Chtf18*-null male mice exhibit both premature homologue disjunction and decreased DNA crossovers. Thus, involvement of CHTF18 in chromosome cohesion during meiosis is biologically plausible. Loss-of-function mutations in cohesin genes have been described in mice. The phenotype of *Chtf18*-null males is not as severe as those for the *SMC1β* or *REC8* cohesin mouse mutants. Both *SMC1β* mutant males and females are sterile; while *SMC1β-*deficient spermatocytes exhibit pachytene arrest, oocytes from *SMC1β*-deficient females show loss of sister chromatid cohesion in metaphase II [61]. *REC8* mutant male and female mice are also sterile and show severe defects in synapsis, sister chromatid cohesion, and meiotic recombination [50], [62]. Although CHTF18 is not absolutely required for meiotic recombination, it may serve as functional link between DSB repair and crossover formation in mammals. We propose a model whereby CHTF18 associates or interacts with cohesin proteins to facilitate and maintain linkage of homologues during meiotic prophase I. Our data support a function for CHTF18 downstream of SPO-11 mediated DSB formation, during early stages of RAD51-mediated DSB repair and upstream of MLH1- mediated DSB repair and crossover formation.

In summary, we derived *Chtf18*-null mice and demonstrated that the gene is essential for male meiosis. Our work reveals important new functions of CHTF18 in mammals, and suggests compelling roles for CHTF18 in male fertility and meiosis. Deletion of *Chtf18* leads to a phenotype in which there is significant impairment of spermatogenesis, meiotic defects, and subfertility. These findings closely resemble those found in humans, where the majority of infertile men present with defects more subtle than complete spermatogenic failure. The requirements for *Chtf18* in mammalian spermatogenesis demonstrated above suggest that malfunctioning of CHTF18 may be a cause of oligospermia and infertility in men. Hence, this work provides an important framework for future studies, which may elucidate the functions of CHTF18 in mammalian meiosis and fertility, and may ultimately shed more light on the processes of DSB repair and chromosome cohesion.

## Materials and Methods

### Derivation of *Chtf18^−/−^* mice and *Chtf18^flox/−^; TNAP* Cre mice

A 129SV mouse BAC library was screened by PCR and colony hybridization to obtain the *Chtf18* genomic clone. Fragments of genomic DNA were then amplified by PCR from the *Chtf18* clone and subcloned into the PND1 plasmid (given by G. Radice, Jefferson Medical College) to construct the PND1-*Chtf18* targeting vector. Following electroporation and selection of cells, targeted clones were enriched by culture in G418. Approximately 300 surviving colonies were isolated, expanded, and screened for homologous recombinants by Southern blot analysis and PCR. Cells from three correctly targeted clones were expanded further, analyzed for a normal karyotype, and injected into C57BL/6 blastocysts, yielding 19 highly chimeric (≥90%) male mice. The male chimeric mice were mated to C57BL/6 female mice, resulting in successful germline transmission of the *Chtf18^flox^* allele. Mice carrying this allele were then mated with transgenic Cre mice under the control of the E2A promoter [27]. The resulting heterozygotes were bred to homozygosity to generate *Chtf18*-null mice. To derive *Chtf18^flox/−^*; TNAP Cre mice, mice heterozygous for the *Chtf18^flox^* allele were bred with transgenic Cre mice under the control of the germ-cell specific promoter tissue non-specific alkaline phosphatase (TNAP) [28] following FLP-mediated excision of the neomycin resistance cassette *in vivo*.

### Western blot analysis

For protein analyses of mouse testes, 50 mg of total protein were electrophoretically separated by 4–12% SDS-PAGE, and transferred to polyvinylidene difluoride membranes (Millipore Co., Bedford, MA). Membranes were blocked (Tris-buffered saline solution containing 5% nonfat dry milk and 0.1% Tween 20 [TBST]), and then incubated with IgG-purified mouse CHTF18 antibody (0.31 mg/ml) [23] at 4°C overnight. The blots were washed in TBST and incubated with a goat anti-rabbit immunoglobulin conjugated to horseradish peroxidase (0.2 mg/ml, Jackson ImmunoResearch Laboratories, Inc., West Grove, PA) for 1 h at room temperature. After washing, the CHTF18 protein was detected with Super Signal chemiluminescent substrate (Pierce, Rockford, IL).

### Sperm concentration

The caudal epididymides of wild-type and *Chtf18* ^−/−^ adult mice were dissected, and their sperm content was released into PBS. Sperm number and concentration were determined using a hemocytometer. Statistical analysis was performed using the Student's t-test.

### Fertility assessment

The number of offspring from wild-type females bred with 3 *Chtf18* ^−/−^ and 3 wild-type male mice over a five month period was documented. Each male was paired with two wild-type females, and the total number of pups from 12 pairs of wild-type females was recorded. Statistical analysis was performed using the Student's t-test.

### TUNEL assay

Testes from wild-type and *Chtf18* ^−/−^ mice were fixed in 4% paraformaldehyde and embedded in paraffin. TUNEL assays were performed with the *In Situ* Cell Death Detection Kit, Fluorescein (Roche Applied Science, Indianapolis, IL) according to the manufacturer's instructions. Two hundred seminiferous tubules from 3 mice of each genotype were counted. Only cross sections and tubules containing at least one apoptotic cell were counted.

### Histology, surface spread nuclei, and immunofluorescence

For histology, testes from adult male mice were fixed in Bouin's solution, embedded in paraffin, sectioned, and stained with hematoxylin and eosin. For germ cell counts, testes from postnatal day 3 mice were fixed in 2% paraformaldehyde, embedded in paraffin, sectioned, and stained with anti-DDX4/MVH antibody (rabbit, Abcam, 1∶250) and DAPI. MVH-positive cells were counted in at least 250 tubules from four different mice per genotype. Surface spreads of spermatocyte nuclei were prepared as previously described [63], [64]. Briefly, mouse testes were removed, seminiferous tubules gently minced with tweezers in DMEM, and cells mechanically separated. The cellular suspension was then spun to pellet cellular debris, and the nuclear suspension was pipetted onto slides. Slides were then fixed for 3 minutes each in freshly prepared 2% paraformaldehyde in PBS containing 0.03% SDS, and in 2% paraformaldehyde alone. Slides were rinsed three times for 1 minute each in 0.4% PHOTO-FLO 200 solution (Eastman Kodak Company, Rochester, NY), dried, then blocked in TBST containing 10% goat serum. Slides were then incubated with primary antibodies for 1 hour at 37°C or overnight at 4°C. Primary antibodies used for immunofluorescence were as follows: anti-SYCP2 (guinea pig, 1∶100), anti-SYCP1 serum 458 (rabbit, 1∶500), anti-SYCP3 (rabbit, Abcam, 1∶200) or anti-SYCP3 (mouse, 1∶1 provided by R. Jessberger, Dresden University of Technology, Dresden, Germany), anti-γH2AX (rabbit, Millipore, 1∶500), anti-RAD51 (rabbit, Calbiochem, 1∶400), anti-CREST (human Immunovision, 1∶100), anti-MLH1 (mouse, BD Pharmingen, 1∶50). Metaphase spreads were stained with Giemsa.

### Mice

All experiments involving mice were approved by the Institutional Animal Care and Use Committees at the University of Pennsylvania and Drexel University College of Medicine.

### Statistical analysis

The data comparing testis size, caudal epididymal sperm concentration, and number of offspring for *Chtf18* ^−/−^ mice and *Chtf18* ^+/+^ controls were subjected to the Student's t-test. Results from expected/observed ratios of *Chtf18* ^+/+^, *Chtf18* ^+/−^, and *Chtf18* ^−/−^ embryos were analyzed by analysis of variance (ANOVA). Germ cell and immunofluorescence focus counts were analyzed using the Chi square test, Fisher's exact test or Student's t-test. All data were expressed as mean ± standard error of the mean (SEM), and p values<0.05 were considered statistically significant. Values were calculated using Prism 4.0 for Macintosh (GraphPad Software, Inc., La Jolla, CA).

## Supporting Information

Figure S1 Breeding strategy to derive *Chtf18* ^flox/−^ TNAP Cre mice (cKO) mice.
(TIF)

Figure S2 The average number of MLH1 foci is significantly decreased in *Chtf18* ^−/−^ spermatocytes that do not lack foci compared to wild-type spermatocytes (23.82 and 22.17 for four *Chtf18* ^−/−^ and four wild-type 21 day old males, respectively, p<0.0001 using Student's t-test).
(TIF)
