# Supplementary figures and images for: Disruption of Chtf18 Causes Defective Meiotic Recombination in Male Mice
Source: PLoS Genet. 2012 Nov 1;8(11):e1002996. doi: 10.1371/journal.pgen.1002996 (PMC3486840; doi:10.1371/journal.pgen.1002996)

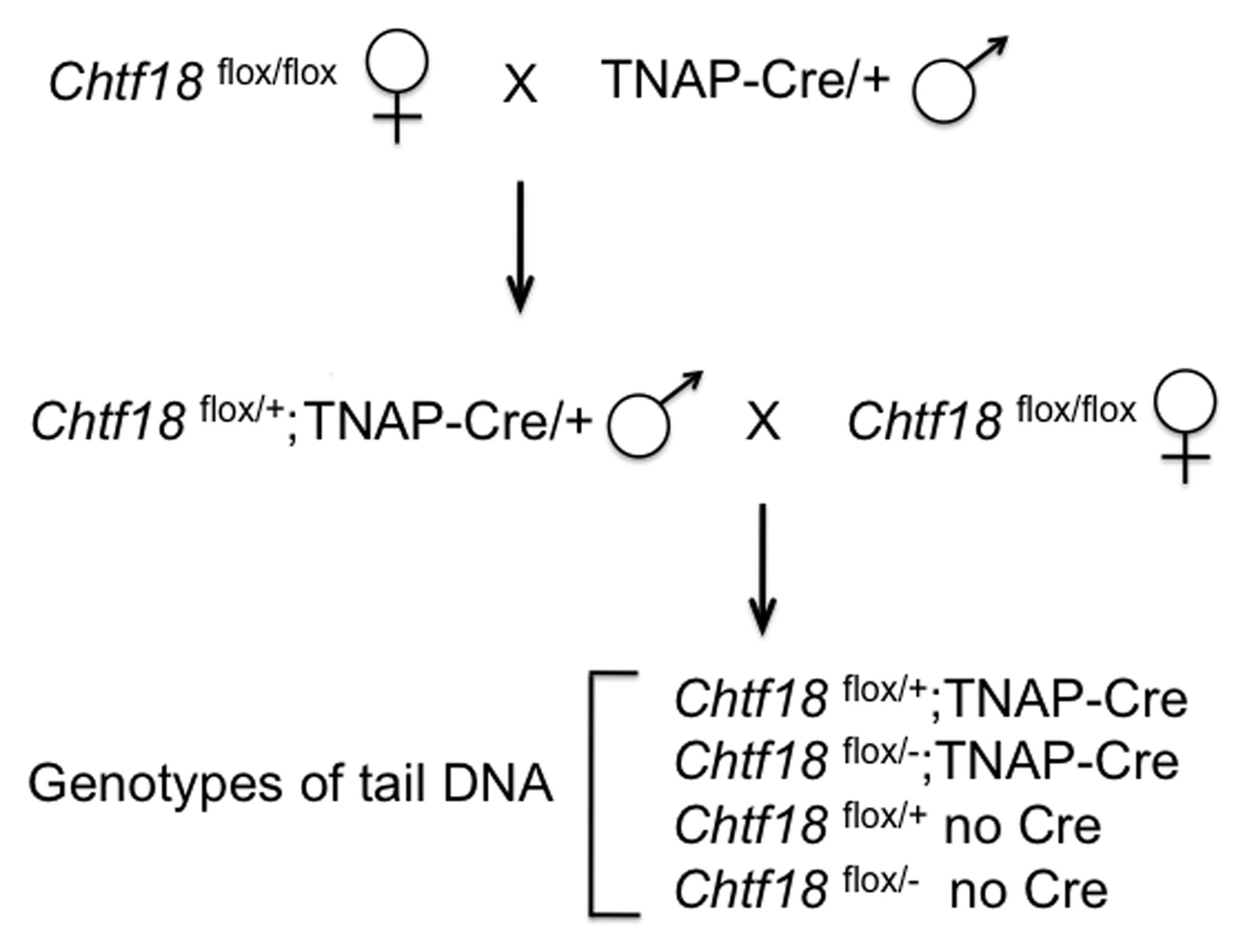

Supplement: Figure S1 — Breeding strategy to derive Chtf18 flox/− TNAP Cre mice (cKO) mice. (TIF) [file pgen.1002996.s001.tif]

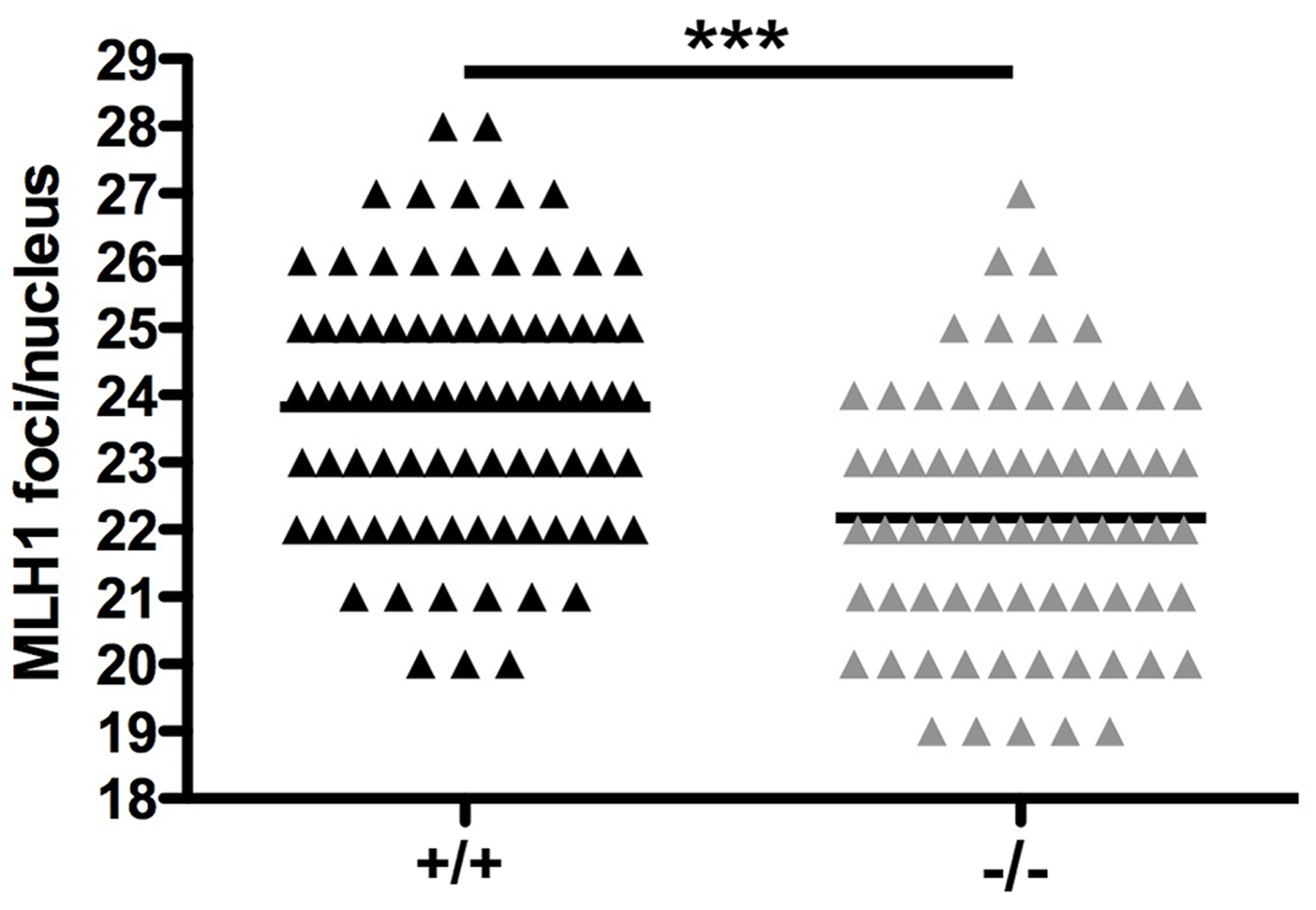

Supplement: Figure S2 — The average number of MLH1 foci is significantly decreased in Chtf18 −/− spermatocytes that do not lack foci compared to wild-type spermatocytes (23.82 and 22.17 for four Chtf18 −/− and four wild-type 21 day old males, respectively, p<0.0001 using Student's t-test). (TIF) [file pgen.1002996.s002.tif]
